# Process strategy to fabricate a hierarchical porosity gradient in diatomite-based foams by 3D printing

**DOI:** 10.1038/s41598-019-55582-0

**Published:** 2020-01-17

**Authors:** I. Capasso, B. Liguori, L. Verdolotti, D. Caputo, M. Lavorgna, E. Tervoort

**Affiliations:** 1 ACLabs Applied Chemistry Labs, Department of Chemical, Materials and Production Engineering, University of Naples Federico II, P.le Tecchio 80, Naples, Italy; 2 Institute of Polymers, Composite and Biomaterials, National Research Council, Naples, Italy; 3 Complex Materials, Department of Materials, ETH Zürich, 8093 Zürich, Switzerland

**Keywords:** Nanoscale materials, Techniques and instrumentation, Materials science

## Abstract

Motivated by the hierarchical micro and nanoscale features in terms of porosity of diatomite, the production of ceramic-graded porous foams with tailored porosity, obtained by using it as raw material, has been proposed. The main challenge during the foam-production process has been the preservation of diatomite nanometric porosity and the addition of other levels of hierarchical porosity. The coupled use of two techniques of direct foaming (chemical and mechanical), combined with the use of 3D printing inverse replica method, assured the achievement of porosity of, respectively, microscopic and macroscopic dimensions. Optical and scanning electron microscopies have been performed for an in-depth characterization of the final microstructure. XRD analysis has been carried out to check the influence of sacrificial templates on the matrix mineralogical composition. The porosity of the diatomite-based foams has been investigated by means of nitrogen-adsorption analysis and mercury-intrusion porosimetry. The experimental tests confirmed the presence of different porous architectures ranging over several orders of magnitudes, giving rise to complex systems, characterized by hierarchical levels of porosity. The presence of porosity of graded dimensions affects the final mechanical performances of the macroporous diatomite-based foams, while their mineralogical composition does not result to be affected by the addition of templates.

## Introduction

The fabrication of foam materials with a porosity gradient (functionally-graded porous materials-FGPMs) is widely desired from scientists in order to achieve an enhanced performance (3D functionally-graded properties), mainly due to their low density, high strength and specific functional properties. FGPMs are innovative materials suitable for specific and advanced functions, in which a spatial gradation in structure and/or composition leads to tailored properties^1^. For this reason, these materials find application in a broad range of high-tech fields such as energy, building, aerospace, filtration and bioengineering. In many of these applications, hierarchical structures with pores of distinct sizes are desired to achieve a proper balance between conflicting properties^2^. Generally, graded porous materials are materials in which the properties vary in a given direction (aligned porosity: unidirectional, lamellar or lotus type)^3^ and this is the major advantage compared to other composite materials. Several processing routes have been explored to fabricate FGPMs (mainly ceramic materials based) including templating, etching, freeze casting^3^, microwave sintering processes and compression-molding salt leaching^4^. In the last years, apart from the aforementioned conventional processes, the use of advanced techniques such as additive manufacturing, which permits the fabrication of complex geometries with high accuracy, to make graded porosity (with predicted distributed porosities) and compositions, still remains a challenge^5^. Furthermore, this is even more a challenge if the “starting material” itself contains an intrinsic gradient porosity, obtained through physical and chemical blowing reactions and in specific environmental conditions^6,7^.

Improvements in conventional processing methods and the development of innovative fabrication approaches are required because of the increasing specific demands on properties and morphology (cell size, size distribution and interconnection) for these materials, which strictly depend on the application considered^8^. Accordingly, combining a new fabrication method (3D printing) with foaming techniques can be considered an innovative approach in the production of graded porous ceramic foams^9–11^. For example, the additive manufacturing (AM) of ceramic foams has become an attractive field of research that has the potential to disruptively change the way complex-shaped bodies are fabricated^12^. An even more enticing feature that has only recently started to be explored is the use of AM technologies to create materials with locally-tuned chemical compositions and intricate microstructures that are not accessible by conventional processing routes^13^. However, the use of this kind of processing technologies for ceramic materials is challenging because they are not easy to process considering their processing requirements (in terms of feedstock and/or sintering)^12^. Consequently, an indirect inverse-replica approach where (macro-) porous structures (templates) are printed and the ceramic slurry is cast into the template cavity, can overcome some of the limitationsin the physical, functional, and geometrical characteristics of components produced of the other AM techniques^14^ especially of the direct printing. In this way, in fact, templates and molds can be produced with high levels of accuracy using common polymers, taking advantage of freedom and shape possibilities provided by this technique. Moreover, the use of an indirect inverse-replica approach allows to avoid limitations, in terms of homogeneity and microstructure control of the final ceramic part, typical of other AM techniques, by filling directly the templates with suitable slurries^15^. Finally, in this case, the rheological properties required to the slurry are much less stringent compared to those required in the case of the direct printing in which the rheology of the inks definitely plays a key role. In particular, in this paper, stereolithography has been selected as 3D-printing technique for the production of the polymeric sacrificial templates needed to add macropores to diatomite based foams.

So, taking inspiration from these considerations, the aim of this paper is the design and synthesis of sustainable functionally-graded porous ceramic materials with hierarchical and tailored porosity. These are obtained by starting from a natural raw material, diatomite, which is similar to seashells, trying to fully exploit its intrinsic features. Diatomites, opokas, tripolis, spongiolites, and radiolarites form a group of sedimentary silica rock consisting predominantly of opal and cristobalite. The interest shown in the study of siliceous rock is largely due to their useful properties^16,17^. Recently, diatomite was proposed as active support to obtain hierarchically porous nanocomposites with interesting application in environmental field^18–20^. The addition of other levels of porosity, with varying sizes, to the nano porosity already present and typical of diatomite^21,22^, leads to a bio-inspired final product with hierarchical and graded porosity that can be properly tuned according to the final properties desired for the material.

For the design and the production of the solid ceramic matrix, starting from diatomite, the so-called alkali-bonded ceramics or chemically bonded ceramics (CBCs), have been selected as reference materials, considering their ever increasing number of possible applications^23–26^. CBCs are produced through a chemical reaction, consisting of a polycondensation of silica phases dissolved in a strong alkaline environment at low temperature, in contrast to traditional ceramic materials that are usually produced using fusion or sintering processes at elevated temperature. The complete production process of the diatomite-based foams is well-known and is accurately described in our previous works^6,7^, where it has also been demonstrated that the chemical–physical properties and density of the resulted foams can be tailored using the proposed aproach. In this manner, the innovative manufacturing process proposed in the present paper guarantees the presence of both microscopic- and macroscopic-scale porosity, preserving at the same time the nanoscale porosity of the starting diatomite, leading to ceramic foams with graded and tunable porosity.

## Results

### Production of macro-pores by 3D printing inverse replica method

**Figure 1:**
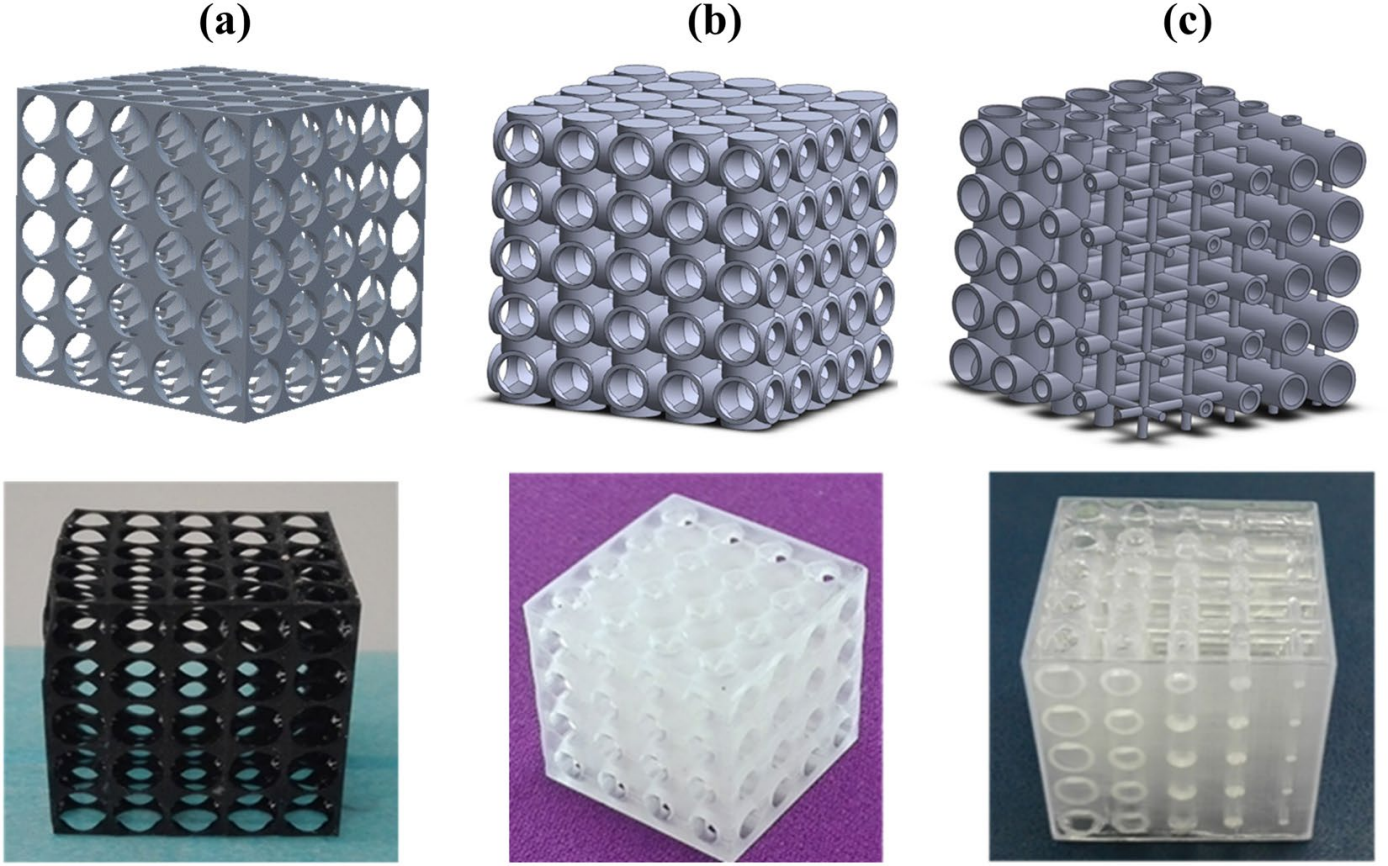
3D rendering and printed (lower part) of the three different sacrificial templates.

Three different geometries of sacrificial templates have been selected and printed in order to investigate the influence of the template geometry on the final microstructure and porosity of the diatomite based foam (Fig. 1).

The printed models replicate exactly the computationally designed desired macro-scale pore sizes and architectures; the solids struts in the polymeric scaffold will constitute the macropores in the inverse replica foam structure. In particular, the template reported in Fig. 1(a) is characterized by the presence of a regular geometry with round openings of fixed dimensions (3 mm), the template reported in Fig. 1(b) shows a regular geometry with fixed dimensions of the round openings (3 mm) and square channels (2 mm), while the sacrificial template reported in Fig. 1(c) is characterized by a varying dimension of round openings, ranging from 4 to 0.5 mm, along two different directions.

**Figure 2:**
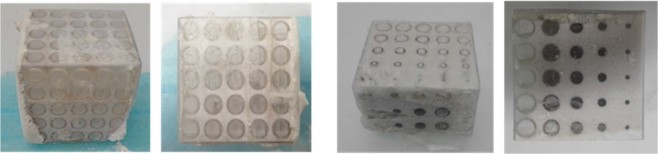
Sacrificial templates impregnated with diatomite-based slurry.

Each sacrificial template has been impregnated pouring the diatomite-based slurry in all their cavities (Fig. 2) and then the obtained systems cured at 40 °C and at room humidity for 24 hours.

**Figure 3:**
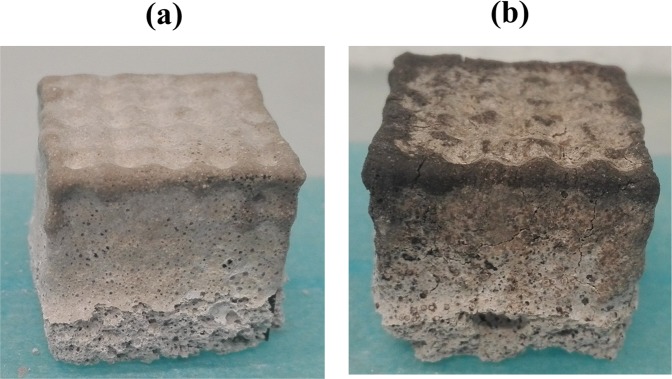
Sacrificial template impregnated with diatomite-based foam after curing (**a**) and after template removal process (**b**).

**Figure 4:**
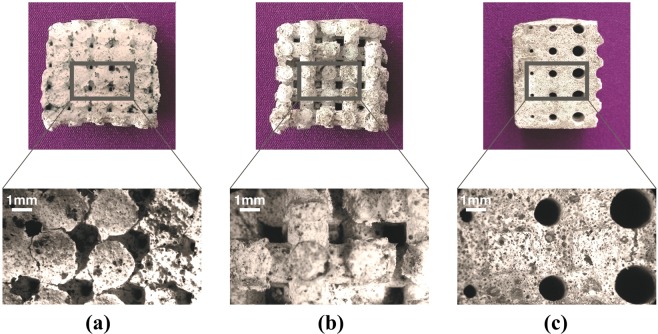
Diatomite-based foam inverse replica obtained after the complete template removal and corresponding stereomicroscopic images.

It is possible to notice that no shrinkage and no evidence of swelling are present after the curing time (Fig. 3a), indicating that the consolidation process was not hindered inside the polymeric template. After the thermal treatment at 500 °C for 6 hours, ST_DHCF resulted to be still solid and did not show presence of significant or critical cracks or fractures (Fig. 3b). This proves that the template removal process was completed successfully and that the process parameters (heating rate and temperature) have been properly selected. The final lattices obtained after the impregnation of the three different template architectures, followed by the template removal, are shown in Fig. 4.

All the lattices of the produced foams are effectively precise and accurate inverse replica of the 3D printed designed structures (Fig. 1, lower part). Moreover, thanks to the complete and homogeneous infiltration, the diatomite-based slurry was able to penetrate in all the macroscopic openings designed in the template CAD models. The systematic and periodic pattern of the macropores, due to the use of polymeric templates, is widely showed (Fig. 4). Looking at the final lattices obtained, also the corresponding stereomicroscopic images are reported in Fig. 4. In particular, it is possible to easily identify the round macropores of about 1 mm left in the foam matrix after the removal of the template (Fig. 4a), the square openings of 2 mm deriving from the square channels present in the template (Fig. 4b) and finally the presence of round pores with different dimensions (from 1 to 3 mm) coming from the architectures of the template (Fig. 4c). All the foams showed the regularity of the macropores structure and no cracks or cavities within the foam matrix can be revealed. After the burning out of the sacrificial template, the millimetric dimension of the macropore was preserved (see Supporting Information).

### Characterization of macroporous diatomite based foams

**Figure 5:**
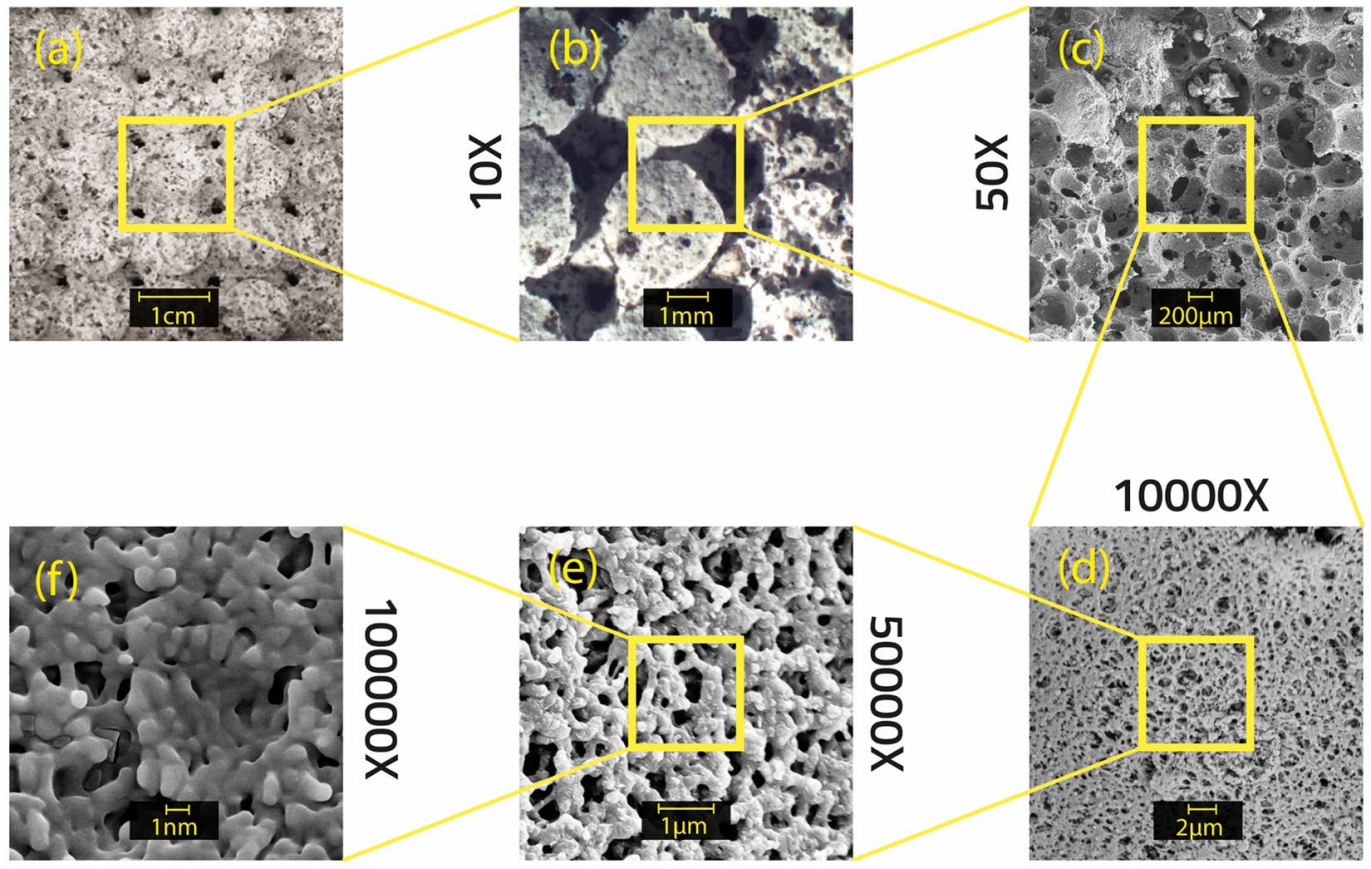
SEM images of STa_DHCF sample at different magnifications ranging from centimeter to nanometer scale.

SEM analyses have been performed also at higher magnifications, in particular on the foam sample obtained infiltrating the template reported in Fig. 1(a), characterized by the presence of a regular geometry with round openings of fixed dimensions (3 mm), which is taken as sample reference for the whole following characterization section and named throughout the text as STa_DHCF, and the corresponding results obtained are reported in Fig. 5. Looking at the microstructure at different magnifications, it results quite evident that the STa_DHCF sample showed an homogenous microstructure characterized by a considerable compactness of the ceramic matrix and by a very fine and small porosity (Fig. 5d), well distributed within the matrix. In particular, looking at lower magnification (Fig. 5c), it is evident the formation of micro and macroporosity due to the presence of both, chemical and physical, foaming agents (as widely described by Liguori *et al*.^7^. It is also possible to identify the presence of a significant porous structure at nanometric scale ranging from 100 to 400 nm. Moreover, looking at the highest magnifications (Fig. 5e,f), it appears quite evident the presence of a sponge-like porous structure that can be related to the consolidation mechanism that takes place and due to the formation of a polysilicate on the surface of diatomite where the reaction of the same diatomite with the alkaline silicate solution used as activator occurred.

Moreover, comparing the micrographs with the results obtained in our previous work^27^ it can be assessed that the thermal treatment due to the template removal process does not affect the microstructure of the foam, in fact no significant changes can be detected in the cellular structure of the foam. This was predictable considering the mainly ceramic nature of the foam matrix and consequently its intrinsic property of high resistance to elevated temperatures. Furthermore this represents a confirmation that the addition of macropores to the already expanded system does not influence negatively the final properties of the diatomite based foam.

**Figure 6:**
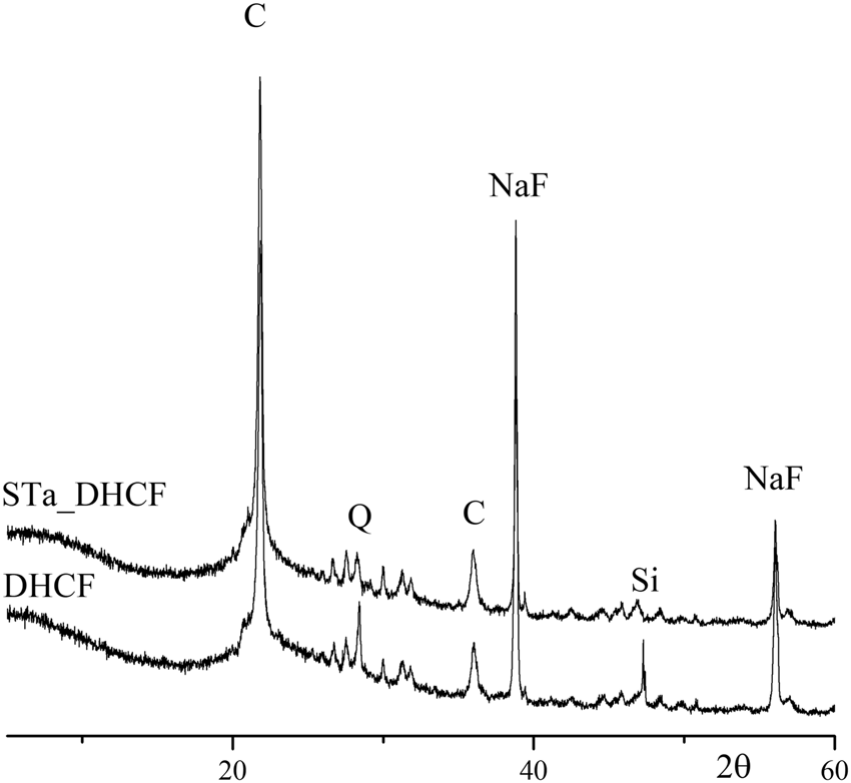
XRD spectra of diatomite-based foam (DHCF) and macroscopic foam sample obtained after template removal (STa_DHCF).

The XRD spectra of diatomite based foam without macroporosity addition and of the macroscopic foam sample STa_DHCF, are reported in Fig. 6. Both XRD patterns showed an amorphous background with a significant amount of crystalline phases: a peak centered approximately at 27° 2θ, related to the quartz, Q (JCPDS card n. 85–0794); two other peaks, at 22° and 36° approximately, reveal that the main SiO_2_ crystalline phase is the cristobalite, C (JCPDS card n. 76–0939).); at 38° and 56°, there are two peaks related to sodium fluoride, NaF (JCPDS card n. 36–1455) that is originated from the reaction between catalyst and sodium. Finally, the peak at 47° is related to the unreacted silicon (JCPDS card n. 75–0589).

Looking at the spectra, it appears evident that the mineralogical composition of the diatomite based foam does not result to be affected at all by the template addition and the following removal process. The main crystalline phases present, in fact, resulted to be the same in both spectra and, consequently, it is not possible to identify any other new peak related to potential mineralogical phases due to the possible interaction between the polymeric template and the ceramic matrix.

**Figure 7:**
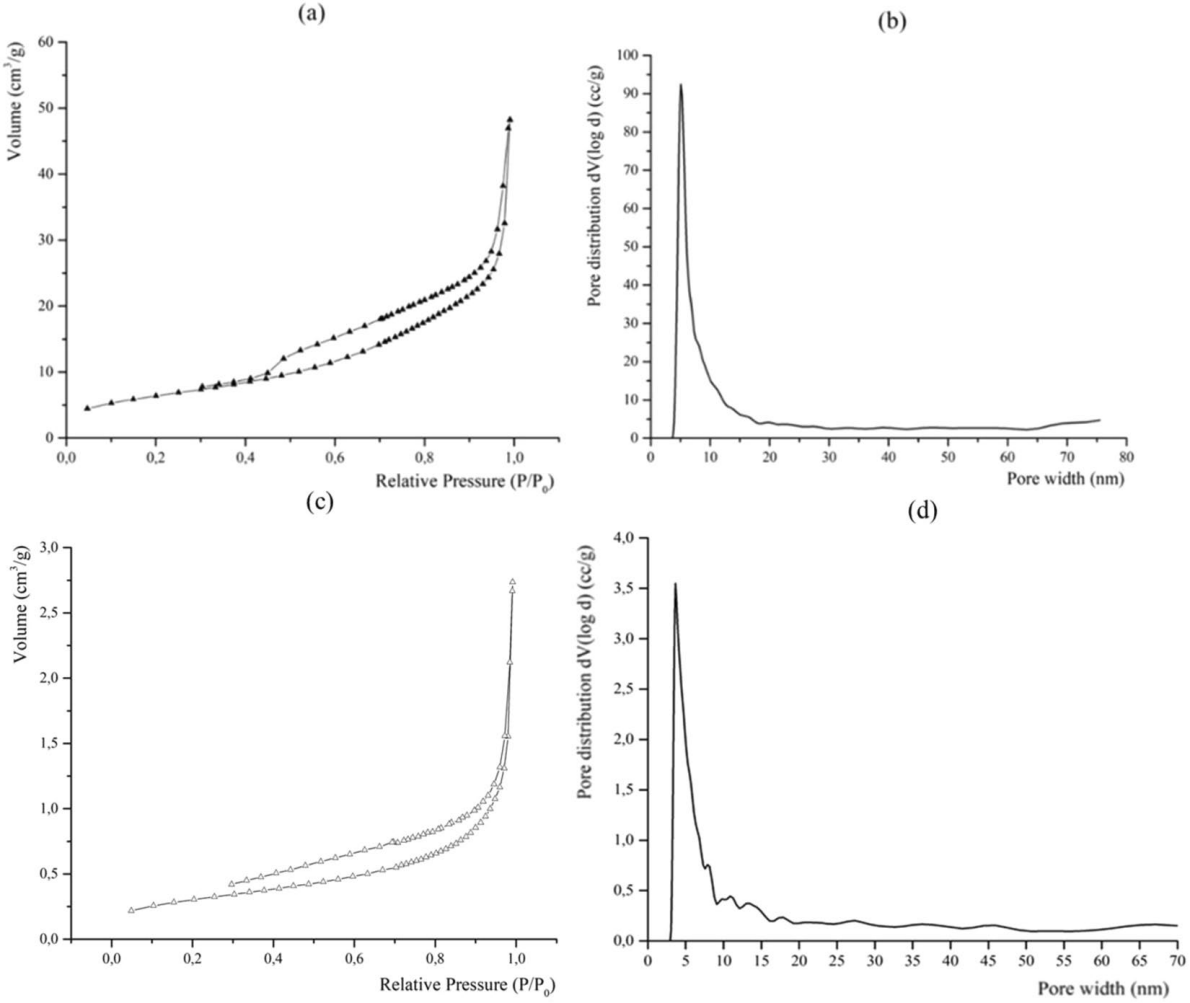
N_2_ gas adsorption isotherms of macroscopic diatomite based foam STa_DHCF. (**a**) and diatomite (**c**) and DFT pore size distribution of STa_DHCF (**b**) and diatomite (**d**).

The results obtained from the N_2_ adsorption analyses performed on the STa_DHCF samples produced are reported in Fig. 7.

According to the Brunauer classification of the adsorption isotherms, the foam sample showed isotherm belonging to the type II. Moreover, the STa_DHCF isotherm resulted to be characterized by the appearance of hysteresis, absent in the isotherm of the raw diatomite reported in Fig. 7c, that can be explained as a consequence of the presence of mesoporosity in the microstructure of the ceramic foam. In particular, in this case, the shape of the hysteresis seems to suggest the existence of mesoporosity with slit-like morphology, typical of pores deriving from the aggregation of flat particles. The pore size distribution obtained using density functional theory (DFT) method for the STa_DHCF sample showed a very narrow and high peak between the values of about 5 and 7 nanometers (Fig. 7b). This means that the most of the pores present in the diatomite-based foam, has dimensions included in that dimensional range. It is a confirmation of the presence of nanoporosity of very small dimensions that derives from the nanopores originally present in the starting diatomite (see Fig. 7d).

In order to characterize also porosity of bigger dimensions, the pore size distribution resulted from the Hg intrusion is reported in Supporting Information. It is possible to deduce that diatomite-based foam showed a monomodal pore distribution located at around 60 μm. Moreover, the values of pores total volume fraction (*x*_*p*_) obtained is equal to 82.05%.

**Figure 8:**
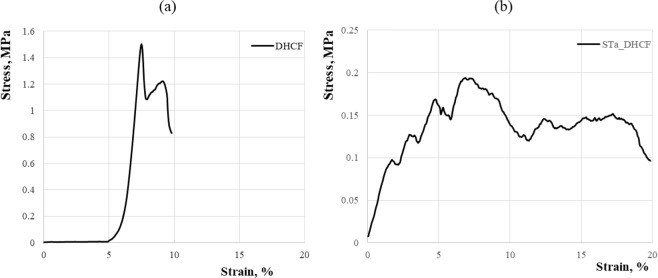
Stress-strain diagrams of diatomite-based foam (**a**) and diatomite-based foam after the macroporosity addition STa_DHCF (**b**).

The results of the mechanical tests performed on STa_DHCF samples are reported in Fig. 8(b). The compressive strength values obtained for the three different samples (data not reported) resulted to be very similar, with an average value of 0.203 ± 0.018 MPa. This means that the performed tests can be considered highly reproducible. In Fig. 8(a) the stress strain curve of diatomite foam samples without the addition of macropores is reported^28^ and it can be used as a reference for the evaluation of the mechanical behavior after the addition of macroporosity to the starting ceramic fams. In particular, from the compressive stress–strain curve of STa_DHCF sample, it is possible to notice the presence of a toughening effect probably due to the presence of hierarchical porosity. An initial linear elastic behavior is first observed followed by some strut fractures which correspond to the first slight drops in the stress-strain curves^29^. It is worth notice that the slope of the curve in those first curve segments is basically the same, meaning that the elastic modulus of the sample is almost the same and that the material preserves its own stiffness in this range. This trend in the first part of the stress-strain curve can be related to the typical behavior of foamy materials. In fact, when a foam is compressed at low strains, it deforms in a linear-elastic way because the cell walls at first bend and only for higher strain values they start to deform in non-linear way because of the elastic buckling of their columns or plates^30^. After the linear elastic range, the stress increases as the damage. At the maximum load, macroscopic cracks propagate with a stronger load drop. Finally, densification can occur corresponding to another load increase^29^. It is possible to see that the sample keeps to maintain their stiffness also in this phase. This trend of the mechanical behavior recurs several times before that sample reached its final break and can be explained considering the fact that in the ceramic matrix is present a well distributed porosity of different dimensions. In fact, macropores act like defects or cracks causing the load drop, while the following densification allows the ceramic matrix to support further loads without cracking in a continue alternation that leads to a sort of toughening effect which results to be completely absent in the stress-strain curve of the diatomite based foam obtained without using the 3D printing inverse replica method (Fig. 8a). In this case, in fact, the typical brittle mechanical behavior of ceramic materials resulted to be much more significant. Furthermore, as already mentioned, the presence of diatomite leads to a consolidation process due to the formation of a polysilicate on the surface of diatomite and in particular, the polycondensation of sodium silicate as glassy materials produced bridges between grains and, ensuring higher cohesion, is responsible of the high stiffness noticed^28^ for both samples considered, independently from the presence of macropores within the ceramic foam matrix.

## Discussion

The design and synthesis of ceramic graded porous foams with hierarchical porosity starting from a natural nanoporous material, the diatomite, has been proposed in this paper. In particular, starting from the diatomite as aluminosilicate source, alkali activated ceramic foams have been produced. The system has been foamed using two different techniques of direct foaming, and this approach led to the production of a porosity of micrometric dimensions (100–400 µm) within the diatomite-based matrix which was already characterized by the presence of nanoporosity due to the nanoporous nature of the same diatomite (2–5 nm). The last level of hierarchical porosity (macroporosity) has been added to the diatomite-based foams using the 3D printing inverse replica method and choosing the stereolithography as the additive manufacturing technology for the production of the polymeric sacrificial templates needed. The indirect inverse replica approach allowed to overcome some of the limitations of other AM techniques, especially of the direct printing. In fact, in this process, templates and molds can be produced with high accuracy with common polymers, taking advantage of freedom and shape possibilities provided by this technique and avoiding limitations, in terms of homogeneity and microstructure control of the final ceramic part, filling the templates with suitable slurries. Moreover, in this case, the rheological requirements of the slurry are much less stringent compared to the case of the direct printing. The optical microscopy and SEM analysis confirmed the presence of different porous architectures and confirmed the absence of negative effects of the template addition on the foam microstructure. XRD analysis provided a further confirmation of the stability of the ceramic matrix and its final properties also after the removal of the sacrificial templates. Morever, it has been found out that the presence of this porosity of graded dimensions affects the mechanical properties of the macroporous diatomite based foams causing a toughening effect. Finally, ranging from three orders of magnitudes macro, micro and nano, it is possible to properly tailor the foam porosity according to the final properties desired.

## Methods

Stereolithography has been selected as AM technology for the production of sacrificial templates with a defined and precise geometry, which corresponds to the negative of the geometry desired in the foam sample. In particular, sacrificial templates were designed and converted into stereolitography data using AutoCAD as CAD/CAM software. The model was then imported into a software (Autodesk Print Studio) that sliced it into layers and converted to a code path for the direct 3D printing. The printer used is an Autodesk Ember 3D-Printer, in which the tray holds the liquid resin that the machine turns into 3D parts. It has a clear window made of glass coated with PDMS (a member of the silicone family), through which 405 nm ultraviolet light shines and cures each layer of the print that remains stuck on the printing head. The standard tray of the Ember printer needs at least 50–100 ml resin so that it can be used for printing. The window must allow oxygen permeation in order to form a layer that inhibits polymerization which prevents the print to stick to the window. The resin used to print templates was a standard resin (Autodesk, PR 48) and it was supplied by the printer producers, who provided the following chemical formulation (all percentages are wt/wt):

- Oligomer: Allnex Ebecryl 8210 39.776%, Sartomer SR 494 39.776%
- Photoinitiator: Esstech TPO + (2,4,6-Trimethylbenzoyl-diphenylphosphineoxide) 0.400%
- Reactive diluent: Rahn Genomer 1122 19.888%
- UV blocker: Mayzo OB + (2,2′-(2,5-thiophenediyl)bis(5-tertbutylbenzoxazole)) 0.160%.

An aqueous sodium silicate solution has been used as alkali activator for the consolidation process of the starting diatomite (Diatomite, Celite 545 C), flux-calcined diatomaceous earth, was provided by Celite France) and a catalyst (such as Na_2_SiF_6_) has been added in order to promote the gelification of the entire system. Then, two different techniques of direct foaming have been coupled, one based on chemical reactions with gas and the other one based on a mechanical foaming^6^. The Sodium Silicate water solution (SS) (SiO_2_/Na_2_O = 3,3) was provided by Prochin Italia Srl and has the following chemical composition: Na_2_O 8.15%, SiO_2_ 27.40%. Si metal powder (chemical blowing agent) and Na_2_SiF_6_ were supplied by Merck and Sigma-Aldrich respectively. Vegetable surfactant (physical blowing agent) in water solution (pH = 7) was supplied by Isoltech s.r.l. Italia and used as received after mechanical whipping. Taking into consideration the experimental results already obtained in our previous works^6,7^ the diatomite-based foam has been produced using the following mixture (wt%): 70% of SS, 8.65% of Na_2_SiF_6_, 21.3% of diatomite and 0.05% of silicon powder. Based on 100 parts of the above mixture 12 part of a “meringue” type foam was added. The diatomite-based foam was prepared using simultaneously the silicon powder and the whipped protein as foaming agents, adding both of them to the starting mixture obtained homogeneously dry mixing first the powdered materials (diatomite and Na_2_SiF_6_) and then adding the SS solution.The produced slurry was poured inside the 3D printed sacrificial template and then the so obtained system, namely ST_DHCF, was cured at 40 °C and at room humidity for 24 hours. After curing, the consolidated foam, was thermally treated in order to burn out the polymeric template. The samples were slowly (0.5 °C/min) heated in a furnace (Nabertherm HTC 03/15) at 500 °C for 6 hours, in order to avoid the formation of cracks and fractures inside the foam matrix, caused by the polymer burning out.

In order to investigate the morphology and microstructure and to verify the presence of all the different levels of porosity, optical microscopy (Leica Wild M10, Stereo Microscope) and SEM analysis (SEM, LEO 1530, Zeiss) have been performed on all the produced samples. Also XRD analysis has been carried out to check if the template addition, especially the thermal treatment performed because of the template removal process, affected the mineralogical composition of the starting diatomite based foam. Porosity and specific surface were also evaluated by nitrogen adsorption analysis using a Nova 1000 machine (Quantachrome, USA). Density functional theory (DFT) method was used for determining pore size distribution in the range from micropores to mesopores^31^. Mercury intrusion porosimetry has been performed (Micromeritics, Autopore III) to evaluate macroporosity. Furthermore, the evaluation of pores total volume fraction (*x*_*p*_) was determined as follows:

$${x}_{P}=100\times (1-\frac{\rho }{{\rho }_{0}})$$

in which ρ_0_ is the real density and ρ was the bulk density of the foam, calculated as the mass of the foam divided by its apparent volume. The apparent volume has been geometrically calculated using a caliper (accuracy ± 0.05 mm), while the real density has been obtained performing a He picnometry (Accupyc II 1340, Micromeritics; accuracy 0.03%).

Finally, compressive strength measurements were conducted on cubic ceramic foam samples (10 × 10 × 10 mm^3^) using a universal testing machine (Instron 4411, Instron, USA) equipped with a 1 kN load cell. Measurements were performed in displacement control mode at a rate of 2 mm min^−1^ until the sample fracture was detected in the force–displacement plot. Three cubic samples have been produced and tested.

## Supplementary information

Supplementary Information

## Data Availability

The datasets generated during and/or analysed during the current study are available from the corresponding author on reasonable request.
