# Supplementary material for: Process strategy to fabricate a hierarchical porosity gradient in diatomite-based foams by 3D printing
Source: Sci Rep. 2020 Jan 17;10:612. doi: 10.1038/s41598-019-55582-0 (PMC6969156; doi:10.1038/s41598-019-55582-0)
Supplement: Supplementary file 1 — Supplementary Information [file 41598_2019_55582_MOESM1_ESM.docx]

**Process strategy to fabricate a hierarchical porosity gradient in diatomite-based foams by 3D printing**

I. Capasso^a^, B. Liguori^a,b^, L. Verdolotti^b^, D. Caputo^a^, M. Lavorgna^b^, E. Tervoort^c^

*^a^ACLabs Applied Chemistry Labs, Department of Chemical, Materials and Production Engineering, University of Naples Federico II, P.le Tecchio 80, Naples, Italy*.

*^b^Institute of Polymers, Composite and Biomaterials, National Research Council, Naples, Italy*

*^c^Complex Materials, Department of Materials, ETH Zürich, 8093 Zürich, Switzerland.*

SEM micrographs at low magnifications showed the macropores of millimetric dimensions obtained from the burning out of the sacrificial template.


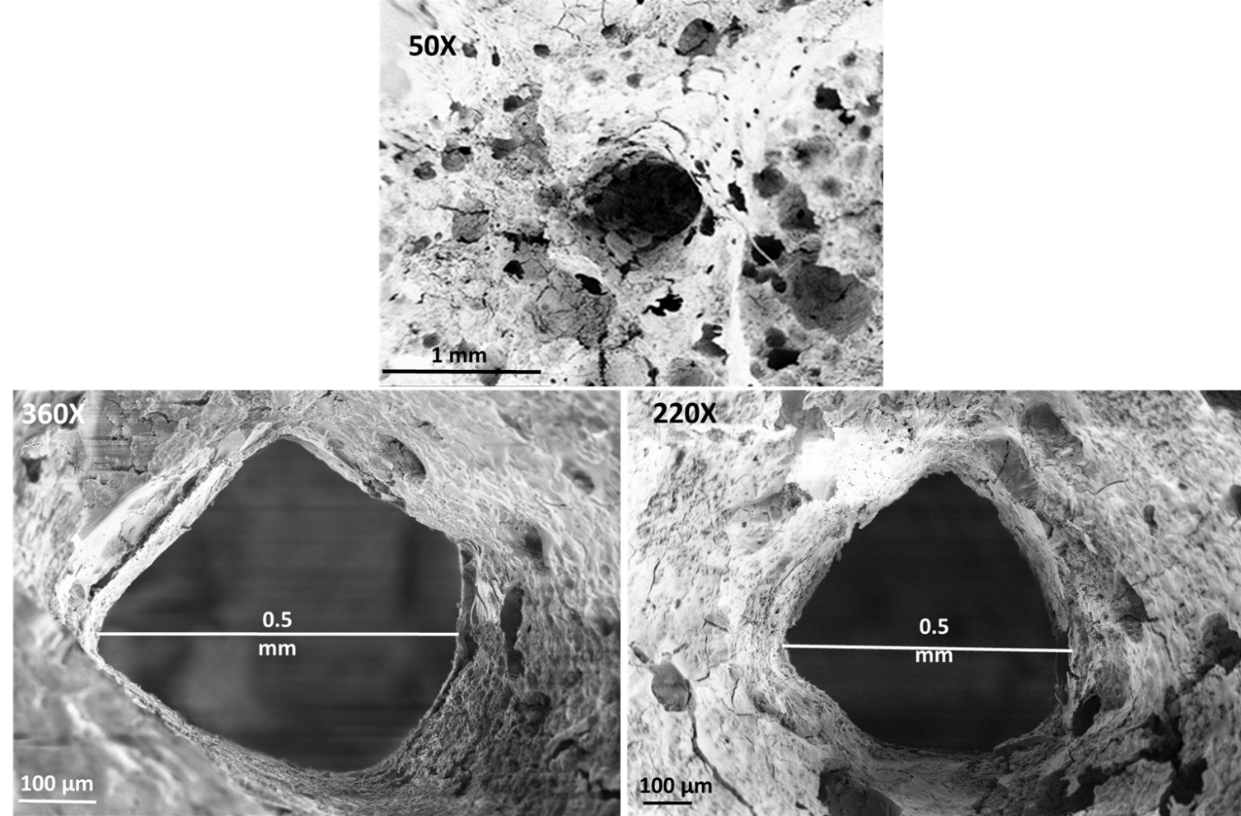


Figure SI_1: SEM images at low magnifications of the macropores deriving from the burning out of the sacrificial template of Figure 1(a)


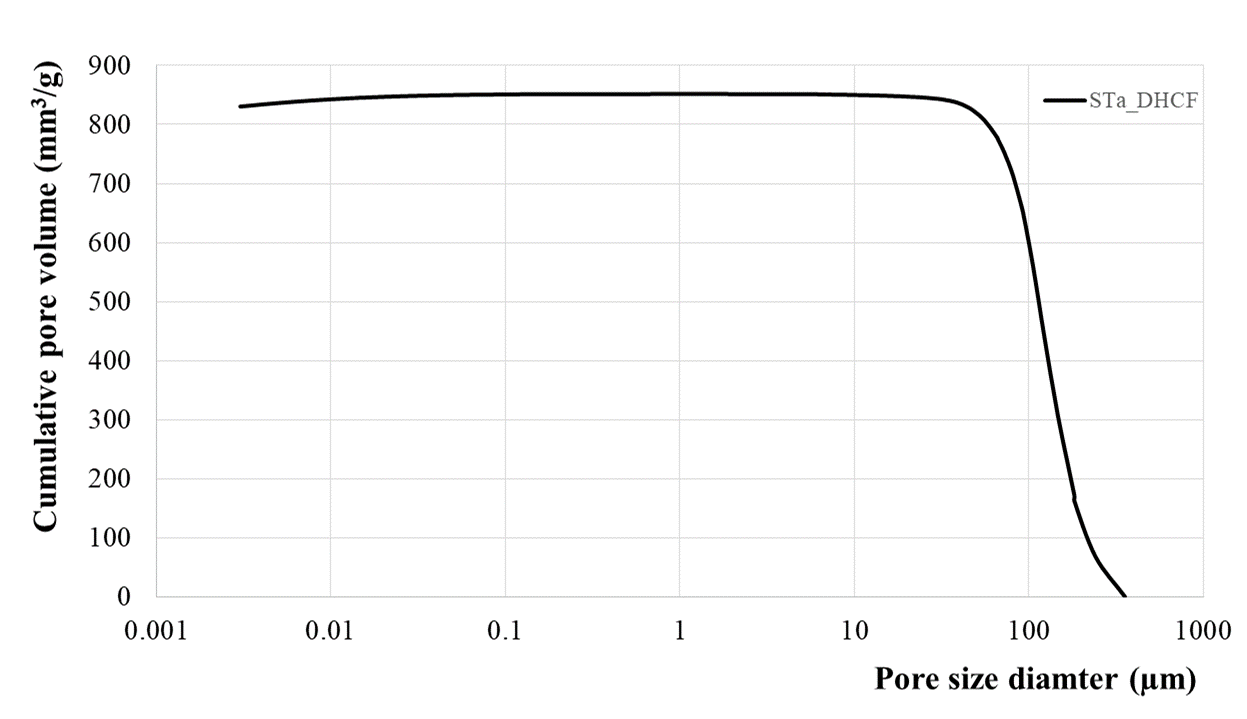


Figure SI_2. Pore size distribution measured by Hg intrusion porosimetry of STa_DHCF sample
